# Human pluripotent stem cell-based models suggest preadipocyte senescence as a possible cause of metabolic complications of Werner and Bloom Syndromes

**DOI:** 10.1038/s41598-020-64136-8

**Published:** 2020-05-04

**Authors:** Kim Jee Goh, Jian-Hua Chen, Nuno Rocha, Robert K. Semple

**Affiliations:** 1 The University of Cambridge Metabolic Research Laboratories, Wellcome Trust-MRC Institute of Metabolic Science, Cambridge, UK; 2 The National Institute for Health Research Cambridge Biomedical Research Centre, Cambridge, UK; 3 Centre for Cardiovascular Science, Queen’s Medical Research Institute, University of Edinburgh, Edinburgh, UK

**Keywords:** Cell biology, Mechanisms of disease, Senescence, Ageing, Embryonic stem cells, Stem-cell differentiation

## Abstract

Werner Syndrome (WS) and Bloom Syndrome (BS) are disorders of DNA damage repair caused by biallelic disruption of the WRN or BLM DNA helicases respectively. Both are commonly associated with insulin resistant diabetes, usually accompanied by dyslipidemia and fatty liver, as seen in lipodystrophies. In keeping with this, progressive reduction of subcutaneous adipose tissue is commonly observed. To interrogate the underlying cause of adipose tissue dysfunction in these syndromes, CRISPR/Cas9 genome editing was used to generate human pluripotent stem cell (hPSC) lacking either functional WRN or BLM helicase. No deleterious effects were observed in *WRN*^−/−^ or *BLM*^−/−^ embryonic stem cells, however upon their differentiation into adipocyte precursors (AP), premature senescence emerged, impairing later stages of adipogenesis. The resulting adipocytes were also found to be senescent, with increased levels of senescent markers and senescence-associated secretory phenotype (SASP) components. SASP components initiate and reinforce senescence in adjacent cells, which is likely to create a positive feedback loop of cellular senescence within the adipocyte precursor compartment, as demonstrated in normal ageing. Such a scenario could progressively attenuate adipose mass and function, giving rise to “lipodystrophy-like” insulin resistance. Further assessment of pharmacological senolytic strategies are warranted to mitigate this component of Werner and Bloom syndromes.

## Introduction

An estimated 425 million people were living with diabetes in 2017, a number projected to increase to 629 million by 2045^1^. 90% of this is accounted for by type 2 diabetes (T2D), intimately related to increasing obesity. Obesity is also associated with a wider constellation of metabolic abnormalities, including insulin resistance (diminished responsiveness of an organism to the hypoglycaemic action of insulin^2^), and metabolic dyslipidaemia, which individually or collectively drive end organ diseases such as polycystic ovarian disease, fatty liver disease and atherosclerosis. Propensity to develop obesity-related metabolic disease shows significant heritability^3–6^, but the mechanistic basis of this has proved hard to tease out, in part because causation cannot be inferred from associations alone.

Severe “obesity-related” metabolic complications are also seen in a heterogeneous group of disorders known as lipodystrophies, in which adipose development or function is impaired. The paradox of obesity-related complications in the face of absent or reduced adipose tissue has been rationalised by invoking “adipose failure” as a unifying feature of lipodystrophy and obesity. According to this notion, metabolic disease ensues when adipose tissue fails to discharge its physiological function as an “energy buffer” which sequesters and stores energy in the face of positive energy balance. Without this buffer other insulin-responsive organs are subjected to lipotoxic stress and ectopic lipid storage. In the case of generalised lipodystrophy adipose failure is absolute, while in obesity it is relative, with the storage capacity of the tissue overwhelmed by excessive demand.

Besides frank monogenic lipodystrophy, in which severe metabolic disease is associated with obvious deficiency of adipose tissue, there are a range of more complex, pleiotropic disorders which feature dyslipidaemic insulin resistance and diabetes with lesser anatomical abnormality of adipose distribution, sometimes only gradually becoming apparent with age. Several of these are caused by defects in genes involved in the cellular response to DNA damage. The canonical examples are Werner Syndrome (WS) and Bloom Syndrome (BS), rare autosomal recessive disorders caused by loss-of-functions mutations in *WRN* and *BLM* respectively^7–9^. *WRN* and *BLM* are ubiquitously expressed RECQ helicases involved in a wide variety of repair processes required to maintain genomic integrity^10^. Patients with WS display clinical features of premature aging, including childhood onset insulin resistant diabetes mellitus, dyslipidaemia, and fatty liver with manifest atherosclerosis from the third decade^7,8,11,12^ as well as early greying, cataracts and cancers. BS patients typically exhibit post-natal growth retardation, a facial ‘butterfly’ rash on sun exposure, defective cellular and humoral immunity, and increased cancer risk, but also are reported to exhibit a high prevalence of diabetes mellitus, dyslipidaemia and fatty liver^13,14^. Both syndromes thus metabolically phenocopy lipodystrophy and obesity, and some reduction of subcutaneous adipose tissue is reported in both syndromes^7,14^. We thus hypothesised that premature adipose failure is at the root of the metabolic disease in these, and perhaps other, DNA damage repair disorders.

Accumulation of cellular DNA damage triggers cellular senescence. Mesenchymal stem cells, one of the major sources of adipose stem or progenitor cells, have been reported to exhibit premature senescence in WS patients^15,16^, while fibroblasts lacking functional *WRN* or *BLM* also show increased tendency to undergo senescence^17,18^. Dysfunctional adipose tissue from obese and/or aged subjects also harbours an increased density of senescent cells^19^, while adipose progenitor cells show diminished ability to differentiate into functional adipocytes^19–21^. Senescent cells exhibit a senescence-associated secretory phenotype, denoting elaboration of proinflammatory cytokines and chemokines such as Interleukin-6 (IL-6), IL-8 and Monocyte Chemoattractant Protein-1 (MCP-1). These have a negative impact on adipose tissue and insulin sensitivity by inducing paracrine senescence in adjacent cells^22–29^. Both genetic and pharmacological studies have established proof of the concept that clearing of senescent cells in adipose tissue can ameliorate systemic metabolism. Increasing understanding of the role played by senescence in adipose tissue in metabolic complications of WS and BS may thus afford new opportunity for precision therapy with senolytic agents in these disorders. Using *WRN*^-/-^ and *BLM*^-/-^ human pluripotent stem cell lines generated using CRISPR/Cas9, this study aimed to model the adipose tissue phenotype seen in WS and BS patients to assess the role played by senescence at different stages of adipose development.

## Results

### Generation of *WRN*- and *BLM*-deficient human embryonic stem cell (ESC) lines

**Figure 1:**
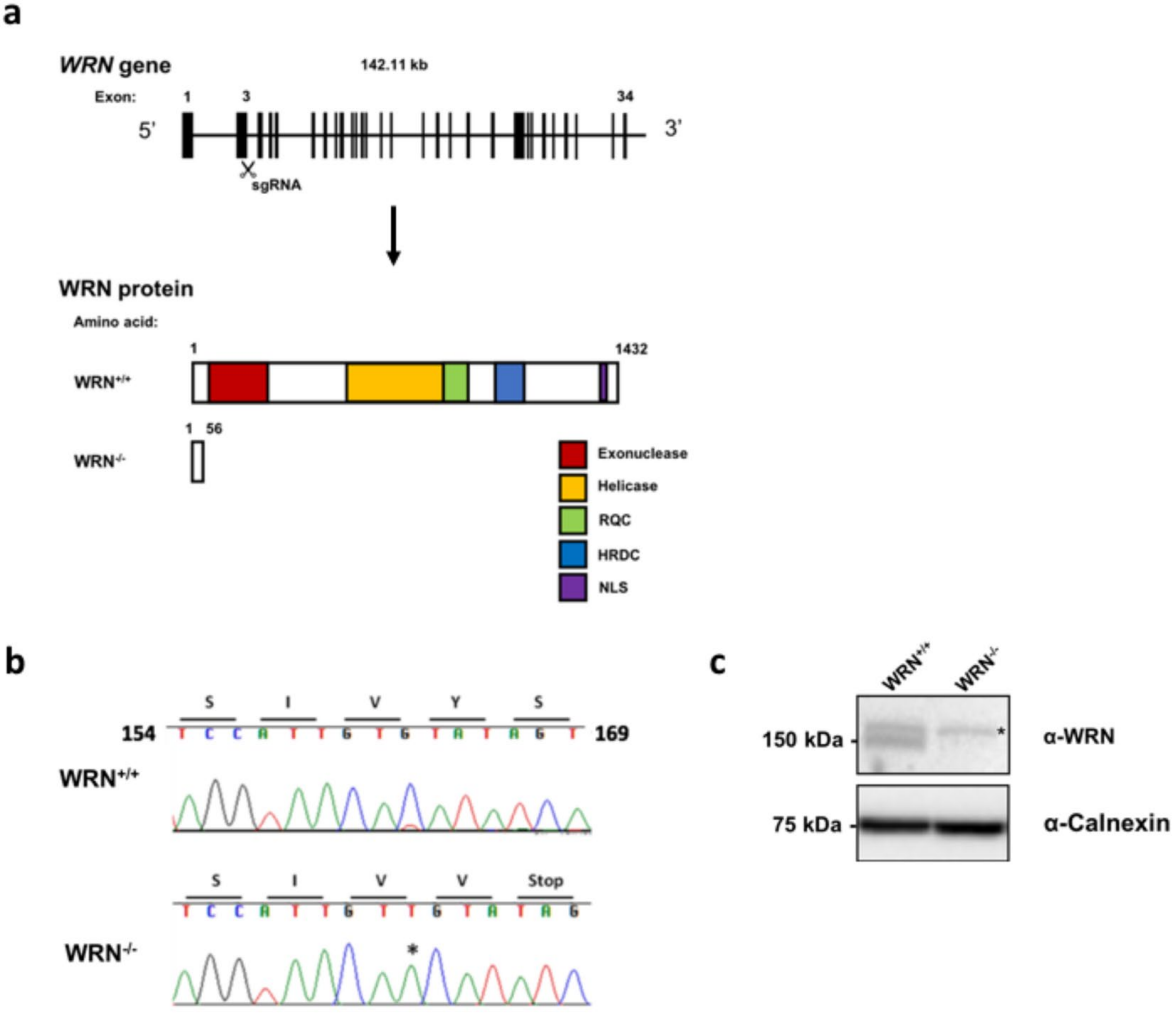
Generation of *WRN*^−/−^ ESCs in the H9 background using CRISPR/Cas9. (**a**) Schematic of the *WRN* locus. Black boxes indicate exons. The sgRNA is designed to target exon 3 of the *WRN* gene. One of the clones with homozygous 1 bp insertion predicted to generate truncated WRN protein was selected for further study, together with one wild type clone. (**b**) *WRN*^+/+^ and *WRN*^−/−^ ESCs were genotyped via Sanger sequencing. *WRN*^−/−^ cells have an 1 bp insertion at position 161, creating a premature stop codon. (**c**) Western blot analysis of WRN^+/+^ and WRN^−/−^ ESCs indicating absence of WRN protein in WRN^−/−^ ESCs. The non-specific band is marked by an asterisk. Antibody used is specific to amino acids 1133–1432 at the C terminus of the WRN protein. Full-length blots can be found in Supplementary figure S4.

To generate a WS cellular model using the well characterised H9 pluripotent human stem cell line, an sgRNA targeting exon 3 of the *WRN* gene was used (Fig. 1a). 24 colonies were picked for screening after targeting, and all but 2 wild-type clones were found to have biallelic gene disruption. No heterozygous clones were observed. Targeting efficiency determined by the percentage of mutated alleles was thus 92%. One wild-type (*WRN*^+/+^) and one mutant clone (*WRN*^−/−^) were selected for further study. The *WRN*^−/−^ clone selected harboured a homozygous 1 bp insertion (c.163insT), creating a premature stop codon (p.Y56Vfs*2) (Fig. 1a,b). The frameshift mutation disrupted WRN protein expression as determined by immunoblotting (Fig. 1c).

**Figure 2:**
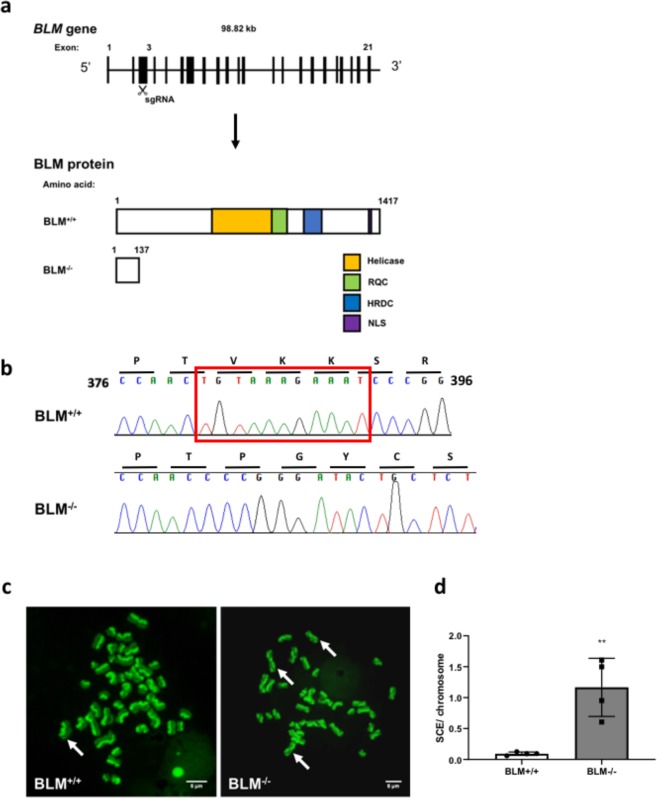
Targeting of *BLM* in H9 ESCs using CRISPR/Cas9. (**a**) Schematic of the *BLM* locus. Black boxes indicate exons. The sgRNA is designed to target exon 3 of the *BLM* gene. The clone with a homozygous 11 bp deletion predicted to generate a truncated version of the BLM protein was selected for further study, together with one wild type clone. (**b**) The genotypes of *BLM*^+/+^ and *BLM*^−/−^ ESCs were assessed *via* Sanger sequencing. *BLM*^−/−^ cells have an 11 bp deletion at position 379 (marked by the red box), resulting in a generation of a premature stop codon. (**c**) Representative images of metaphase chromosomes stained differentially with BrdU and acridine orange with BLM^−/−^ exhibiting “harlequin chromosomes”, a phenotype indicative of Bloom Syndrome. Examples of SCE indicated by white arrows. (**d**) SCE events in 4 metaphase spreads were quantified and expressed as SCE events per chromosome. **p < 0.01. t test.

The BS cellular model was generated using an sgRNA targeting exon 3 of the *BLM* gene (Fig. 2a). Targeting efficiency was 52.1% with only one clone (*BLM*^−/−^) showing biallelic gene disruption, which was due to a homozygous 11 bp deletion (c.381_392del; p.V127Pfs*11) (Fig. 2a,b). Nine clones out of 24 were found to be wild-type. Multiple attempts at immunoblotting failed to detect BLM protein in wild-type or targeted cells, so a functional assay was used instead to validate successful BLM disruption. A hallmark of BS is an increased rates of sister chromatid exchange (SCE), giving a “harlequin-like” chromosomal appearance on karyotyping after labelling of replicating DNA with BrdU^30,31^, which is the basis of diagnostic cytogenetic testing. The chromosomes of the *BLM*^−/−^ clone had a classical harlequin-like appearance consistent with functional deficiency of BLM (Fig. 2c,d).

### Characterization of *WRN*^−/−^ and *BLM*^−/−^ hESCs

All ESC colonies exhibited typical human ESC morphology, with no differences in colony morphology apparent either between *WRN*^+/+^ and *WRN*^−/−^ ESCs or between *BLM*^+/+^ and *BLM*^−/−^ ESCs (Supplementary Figure S1a). All lines stained positively for pluripotent markers OCT4 and NANOG (Supplementary Figure S1b). *WRN*^+/+^, *WRN*^−/−^, *BLM*^+/+^ and *BLM*^−/−^ ESCs could be differentiated into all 3 germ layers, staining positively for SOX17, BRACHYURY and NESTIN, markers for endoderm, mesoderm and neurectoderm respectively (Supplementary Figure S1c). Loss of *WRN* or *BLM* thus does not compromise ESC pluripotency in culture.

**Figure 3:**
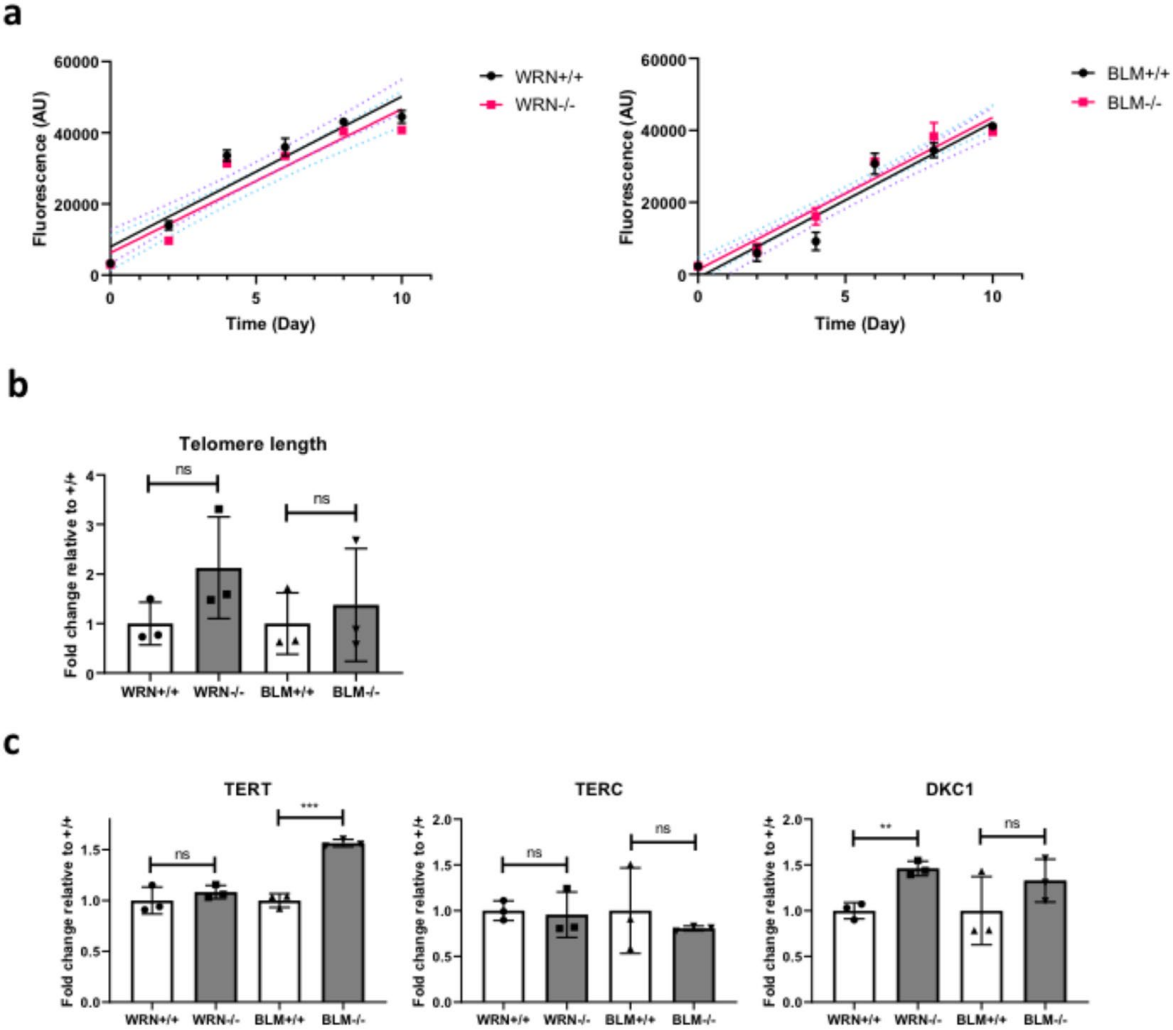
Loss of WRN or BLM does not negatively impact proliferation rates, telomerase expression and telomere length in ESCs. (**a**) Cell proliferation rates of *WRN*^+/+^, *WRN*^−/−^, *BLM*^+/+^ and *BLM*^−/−^ ESCs were assessed using the CyQuant cell proliferation assay. Data represent mean ± SD of three wells. n = 3. Statistical analysis was performed by fitting linear regression lines then testing for gradient equality. The p value was determined to be 0.7368 (*WRN*^+/+^ *vs* *WRN*^−/−^ESCs) and 0.9233 (*BLM*^+/+^ *vs* *BLM*^−/−^ ESCs), indicating that the gradients of the lines were not significantly different from one another. (**b**) qPCR was performed using primers specific for telomeric ends and single copy reference gene 36B4. The relative telomere lengths were then determined by calculating the ratio between the telomeric DNA product and 36B4. Data are represented as means ± SD, n = 3. ns, not statistically significant. (**c**) mRNA from *WRN*^+/+^, *WRN*^−/−^, *BLM*^+/+^ and *BLM*^−/−^ ESCs were extracted for qPCR analysis to determine the expression levels of telomerase complex genes *TERT*, *TERC* and *DKC1*. The housekeeping gene *HPRT* was used as a loading control. Data are represented as means ± SD, n = 3. **p < 0.01. ***p < 0.001, ns, not statistically significant. t test.

Loss of *WRN* or *BLM* also did not affect proliferation rates of ESCs (Fig. 3a). As both *WRN* and *BLM* play important roles in telomere maintenance, telomere lengths were determined using a qPCR-based technique^32^. No significant differences in telomere lengths were found between *WRN*^−/−^ and *BLM*^−/−^ ESCs and their wild-type counterparts (Fig. 3b). Expression of telomerase components telomerase RNA component (*TERC*) and telomerase reverse transcriptase (*TERT*) showed no significant differences between *WRN*^+/+^ and *WRN*^−/−^ ESCs, while dyskerin (*DKC1*) was slightly increased at the mRNA level (Fig. 3c). mRNA expression of TERC and DKC1 did not differ between *BLM*^+/+^ and *BLM*^−/−^ ESCs, but expression of TERT was mildly increased. Taking findings together, we conclude that the loss of *WRN* or *BLM* in ESCs does not impair proliferation nor significantly perturb telomere maintenance in ESCs.

### *WRN*^−/−^ and *BLM*^−/−^ adipocyte precursor (AP) cells show increased senescence

To assess cellular phenotypes potentially relevant to putative adipose dysfunction in WS and BS, *WRN*^+/+^, *WRN*^−/−^, *BLM*^+/+^ and *BLM*^−/−^ ESCs were first differentiated into CD73+ AP cells^33^ (Supplementary Figure S2a). Differentiation efficiency was equally high across all lines (>90%) (Supplementary Figure S2b). AP cells derived from all 4 ESC lines were adherent to plastic and had fibroblastic morphology (Supplementary Figure S2c), indicating that loss of *WRN* or *BLM* does not interfere with the ability of ESCs to differentiate into AP cells.

**Figure 4:**
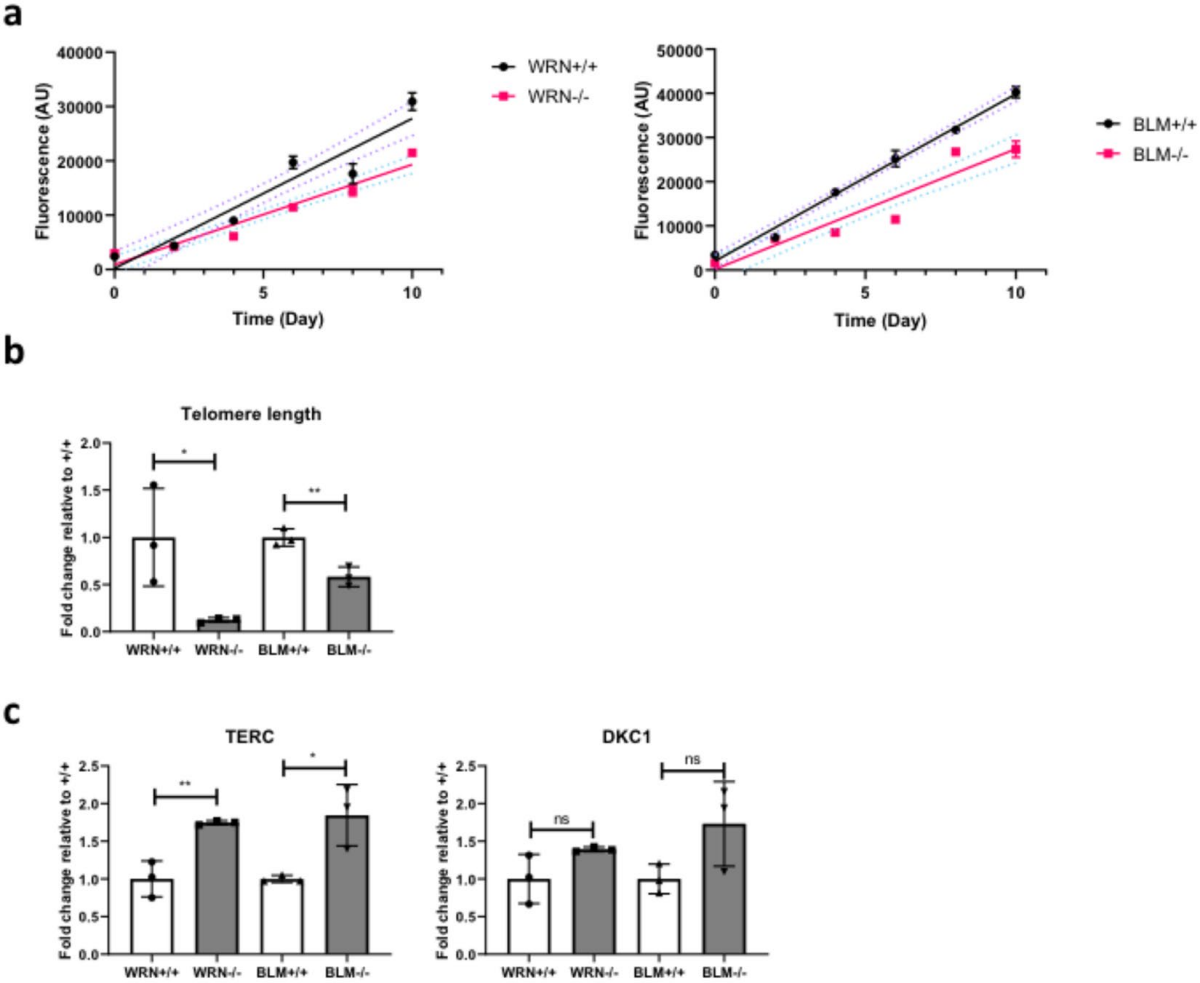
*WRN*^+/+^, *WRN*^−/−^, *BLM*^+/+^ and *BLM*^−/−^ AP cells grow at a slower rate and exhibit defects in telomere maintenance. (**a**) Cell proliferation rates of *WRN*^+/+^, *WRN*^−/−^, *BLM*^+/+^ and *BLM*^−/−^ AP cells assessed using the CyQuant cell proliferation assay kit. Data represent mean ± SD of three wells. n = 3. Statistical analysis was performed by fitting linear regression lines then testing for gradient equality. The p value was determined to be 0.002233 (*WRN*^+/+^ *vs* *WRN*^−/−^ AP cells) and 0.01762 (*BLM*^+/+^ *vs* *BLM*^−/−^), indicating that the gradients of the lines are significantly different from one another. (**b**) The relative telomere lengths were determined by performing qPCR on genomic DNA extracted from *WRN*^+/+^, *WRN*^−/−^, *BLM*^+/+^ and *BLM*^−/−^ AP cells and then calculating the ratio between the telomeric DNA product and *36B4*. Data are represented as means ± SD, n = 3. **p < 0.01, t test. (**c**) mRNA from *WRN*^+/+^, *WRN*^−/−^, *BLM*^+/+^ and *BLM*^−/−^ AP cells were extracted for cDNA synthesis and subsequently analysed via qPCR to determine the expression levels of telomerase complex genes *TERT*, *TERC* and *DKC1*. *TERT* expression levels were however, undetectable and was therefore not shown. The housekeeping gene *HPRT* was used to normalize for cDNA input. Data are represented as means ± , SD n = 3. *p < 0.05, **p < 0.01, ns, not statistically significant. t test.

Proliferation of *WRN*^+/+,^ *WRN*^−/−^, *BLM*^+/+^ and *BLM*^−/−^ AP cells was monitored over 10 days and *WRN*^−/−^ and *BLM*^−/−^ AP cells were both found to proliferate at a slower rate than wild-type counterparts (Fig. 4a). *WRN*^−/−^ and *BLM*^−/−^ AP cells were also found to have shorter telomeres (Fig. 4b). In line with prior studies, *TERT* expression was no longer detectable in AP cells (Data not shown). Expression of *DKC1* was not affected by knockout of *BLM* or *WRN*, while a slight increase in *TERC* expression in both cases is presumed to be insignificant for telomere maintenance given absence of *TERT* (Fig. 4c).

**Figure 5:**
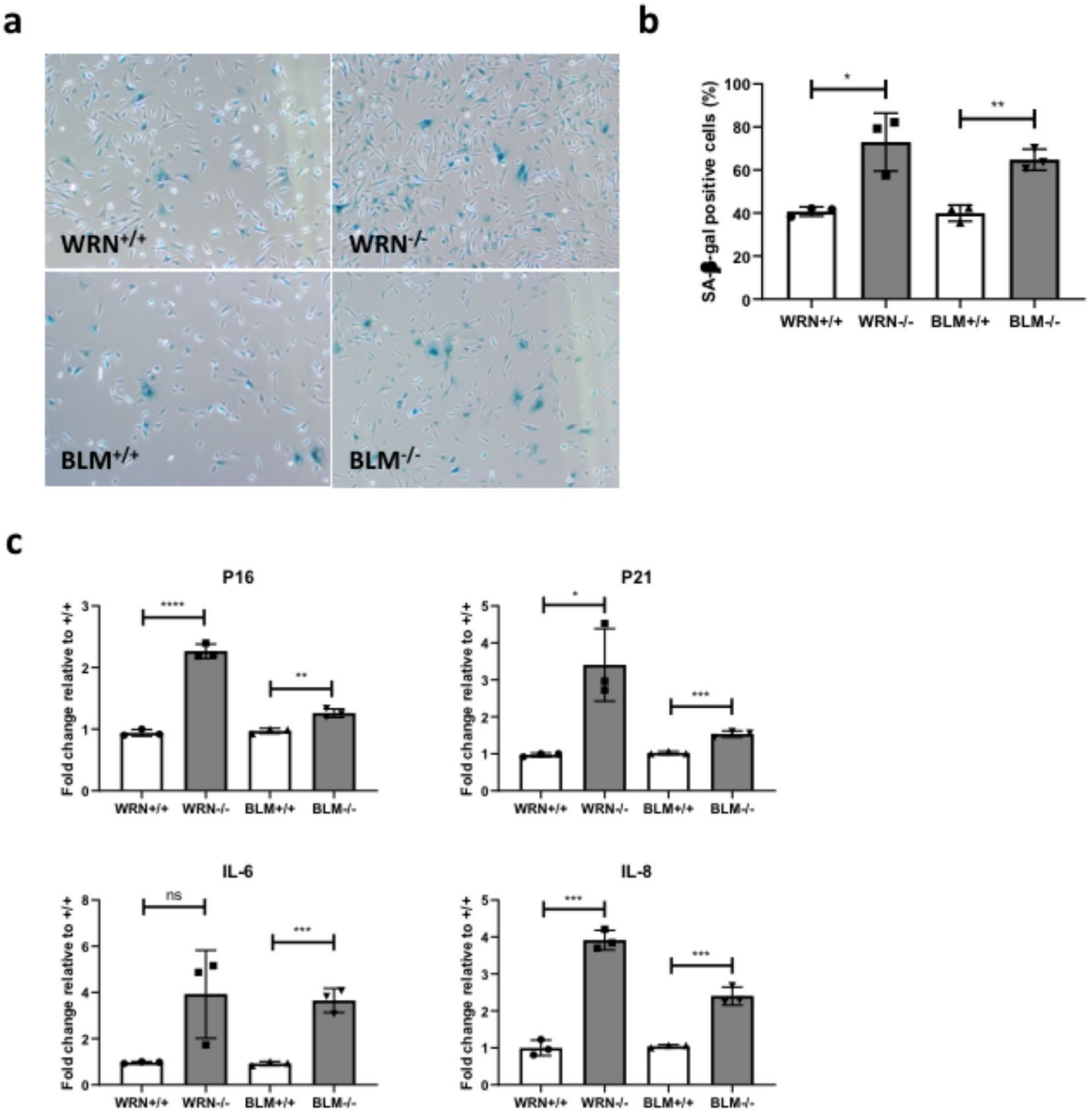
Emergence of premature senescence phenotype in AP cells derived from *WRN*^+/+^, *WRN*^−/−^, *BLM*^+/+^ and *BLM*^−/−^ ESCs. (**a**) Representative bright-field images of SA-β-gal-stained *WRN*^+/+^, *WRN*^−/−^, *BLM*^+/+^ and *BLM*^−/−^ AP cells. Images were captured at 40X magnification. (**b**) Quantification of SA-β-gal-positive cells. Positively stained cells from 5 random fields were quantified and plotted as means ± SD. n = 3. *p < 0.05, **p < 0.01. t test. (**c**) Senescence biomarkers, p16, p21, IL-6 and IL-8, were determined via qPCR in *WRN*^+/+^, *WRN*^−/−^, *BLM*^+/+^ and *BLM*^−/−^ AP cells. *p < 0.05, **p < 0.01, ***p < 0.001. ****p < 0.0001. t test.

AP cells were next stained for SA-β-gal, a commonly used senescence biomarker. *WRN*^−/−^ and *BLM*^−/−^ AP cells contained a significantly higher proportion of SA-β-gal-positive cells (Fig. 5a,b). Expression of senescence markers *p16*, *p21*, Interleukin-6 (*IL-6*) and *IL-8* were assessed by qPCR and all were also upregulated in *WRN*^−/−^ and *BLM*^−/−^ AP cells (Fig. 5c), consistent with activation of senescence gene expression programmes in these cells.

### *WRN*^−/−^ and *BLM*^−/−^ AP cells exhibit attenuated adipocyte differentiation capacity

**Figure 6:**
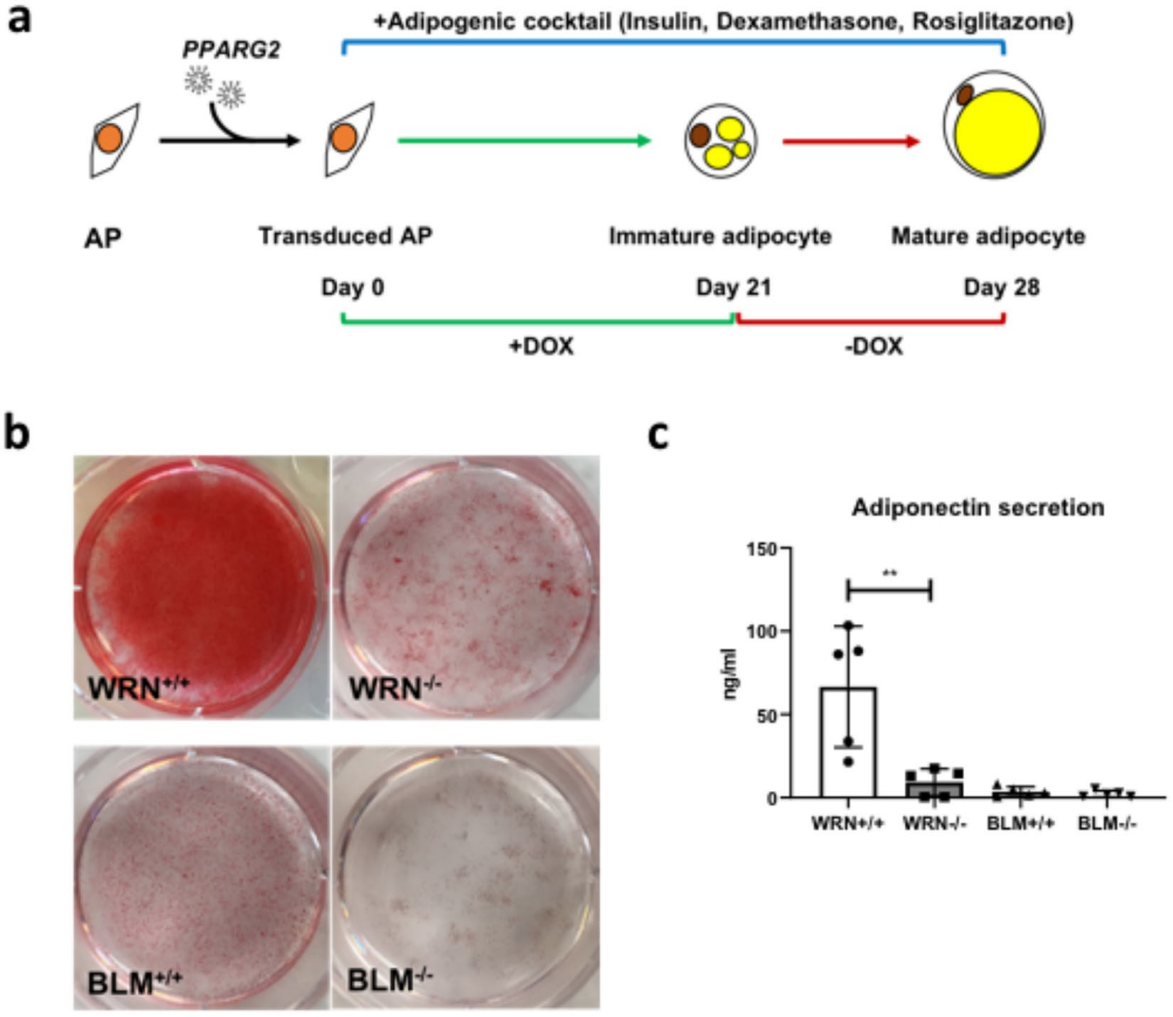
*WRN*^−/−^ and *BLM*^−/−^ AP cells differentiate poorly into adipocytes compared to their wild type counterparts. (**a**) Protocol for differentiating AP cells into mature adipocytes using a combination of *PPARG2* overexpression and an adipogenic cocktail. (**b**) Differentiated *WRN*^+/+^, *WRN*^−/−^, *BLM*^+/+^ and *BLM*^−/−^ AP cells were stained with Oil Red O to allow visualization of lipid droplets as a measure of extent of adipocyte differentiation. (**c**) Media from differentiated *WRN*^+/+^, *WRN*^−/−^, *BLM*^+/+^ and *BLM*^−/−^ AP cells were harvested and ELISA was performed to measure the levels of adiponectin secreted. n = 5. **p < 0.01, t test.

**Figure 7:**
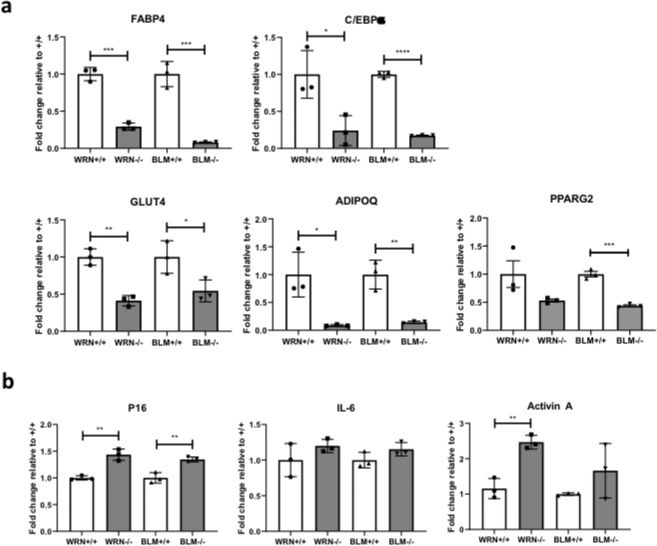
Premature senescence of *WRN*^−/−^ and *BLM*^−/−^ AP cells impairs adipocyte differentiation, possibly through SASP-mediated mechanisms. (**a**) mRNA was extracted from differentiated *WRN*^+/+^, *WRN*^−/−^, *BLM*^+/+^ and *BLM*^−/−^ AP cells and expression levels of adipocyte markers *C/EBPα*, *FABP4*, *ADIPOQ*, *GLUT4* and *PPARG2* were measured via qPCR and normalized to housekeeping gene, *HPRT*. n = 3. *p < 0.05. **p < 0.01. ***p < 0.001. ****p < 0.0001. t test. (**b**) Expression of key senescence and SASP markers were determined via qPCR in differentiated *WRN*^+/+^, *WRN*^−/−^, *BLM*^+/+^ and *BLM*^−/−^ AP cells and normalized to housekeeping gene *HPRT*. n = 3. *p < 0.05. **p < 0.01. ***p < 0.001. t test.

To investigate later stages of adipocyte development, *WRN*^+/+^, *WRN*^−/−^, *BLM*^+/+^ and *BLM*^−/−^ AP cells were next subjected to a previously described adipocyte differentiation protocol^33^ (Fig. 6a). *WRN*^−/−^ and *BLM*^−/−^ AP cells differentiated less efficiently than wild-type counterparts as assessed by intensity of Oil Red O staining (Fig. 6b), and also adiponectin secretion in the case of the WRN knockout experiment, where differentiation of control cells was better (Fig. 6c). mRNA expression levels of adipocyte markers *FABP4*, *CEBPA*, *GLUT4*, *ADIPOQ* and *PPARG2* assessed by qPCR were all found to be significantly downregulated in *WRN*^−/−^ cells relative to *WRN*^+/+^ cells. *BLM*^−/−^ cells were also found to express lower levels of *FABP4*, *C/EBPα*, *GLUT4*, *ADIPOQ* and *PPARG2* compared to *BLM*^+/+^ cells (Fig. 7a). *WRN*^−/−^ and *BLM*^−/−^ cells still showed increased mRNA expression of *p16* (Fig. 7b). Expression of SASP component *Activin A*^34–36^, was also increased in both *WRN*^−/−^ and *BLM*^−/−^ cells relative to the wild-type cells (Fig. 7b), although whether this signal arose from postmitotic differentiated adipocytes or residual senescent APs was not determined. Collectively our data show evidence of increased senescence from early stages of adipocyte development, with attenuated adipocyte differentiation in both WRN and BLM null human cells. This is consistent with the hypothesis that the lipodystrophy-like metabolic complications of WS and BS could arise from premature senescence in the adipocyte precursor compartment.

## Discussion

In recent years there has been growing interest in the role played by senescence in adipose tissue in obesity and/or ageing, with an increase in senescence of preadipocytes shown to decrease adipogenesis and yield lipodystrophy, lipotoxicity and inflammation^19^. Accumulation of DNA damage and/or telomere attrition trigger cellular senescence, and this is a common feature of monogenic disorders of DNA damage repair. Only a subset of this large number of disorders features a “lipodystrophic” pattern of insulin resistance, however, including Werner and Bloom Syndrome. This led us to hypothesise a particular vulnerability to telomere attrition and senescence in the mesenchymal lineage leading ultimately to mature adipocytes.

Faithful *in vivo* models of WS and BS would be the ideal tool for dissection of this phenomenon, however although *Wrn* knockout mice have been generated, to date there are no BS mouse models, as loss of *Blm* caused embryonic lethality^37,38^. Furthermore only when the telomerase component *Terc* was also knocked in *Wrn* knockout mice, and when several generations were allowed to pass, did they display phenotypes resembling WS^39^. This implicated telomere attrition in WS, given the much longer telomeres found in mice than humans. This makes mice challenging from a practical, financial and ethical point of view as models of WS and emphasizes the need for human cells to model human disease.

Here we report successful generation of *WRN*^−/−^ and *BLM*^−/−^ ESCs and isogenic wild-type counterparts using CRISPR/Cas9. *WRN*-null hPSCs have previously been developed either by reprogramming WS dermal fibroblasts into induced pluripotent stem cells^15,40^ or by deleting exons 15 and 16 of the *WRN* gene in H9 and H1 cells^16^. Consistent with our findings, all reports agree on no overt differences between wild-type and *WRN*-null cells in maintenance of pluripotency^15,16,40^. To date, no *BLM*-null hPSCs have been reported although *Blm*-null mouse ESCs were first reported in 2004^41^. This study is therefore the first to successfully model BS in hPSCs. Similar to the *WRN*^−/−^ ESCs, the *BLM*^−/−^ ESCs were capable of maintaining pluripotency and could be propagated over several passages without loss of pluripotency or proliferative capabilities. Although care was taken to utilise control cells that had gone through the same targeting procedure with the same guide RNA, albeit without editing of the target locus, and that had been subject to the same bottleneck of selection, a limitation of our study is that only single clones for each genotype were studied.

Upon differentiation of *WRN*^−/−^ or BLM^−/−^ ESCs into AP cells, premature senescence quickly emerged, as assessed by decreased proliferation, increased SA-β-gal staining, and transcriptional upregulation of senescence markers and SASP components. These findings in WRN^−/−^ cells are consistent with previous reports^15,16,40,42^, which suggest that cells derived from the mesenchymal lineage are particularly susceptible to senescence in the absence of *WRN*^15,43,44^. Whether BS, like WS, also exhibits a segmental, or lineage restricted pattern of premature senescence remains to be determined. More generally, the determinants of this lineage specificity are unclear given that both *WRN* and *BLM* are ubiquitously expressed^45,46^.

*WRN*^−/−^ and *BLM*^−/−^ AP cells showed impaired differentiation into adipocytes compared to wild-type counterparts, in keeping with senescence in the APs, which prior work suggests cells can autonomously induce senescence and dysfunction of otherwise healthy cells through secretion of SASP components such as *IL-6*^47–49^. Indeed this was upregulated in *WRN*^−/−^ and *BLM*^−/−^ AP cells compared to the wild-type. In addition to the *WRN*^−/−^ and *BLM*^−/−^ ESC lines generated using CRISPR/Cas9, we also attempted shRNA-mediated *WRN* or *BLM* knockdown in primary adipose-derived stem cells and the Simpson-Golabi-Behmel Syndrome (SGBS) human preadipocyte line. However, we were unable to sustain these cells in culture long enough for study due to rapidly increased senescence, consistent with observations in the *WRN*- and *BLM*-null ESC-derived APs. This adds to evidence that the lipodystrophy-like metabolic phenotype observed in WS and BS patients may be attributable to senescence in the adipose lineage.

Importantly, proof of the concept that clearance of senescent cells from ageing adipose tissue enhances metabolic state has been established using both genetic and pharmacological approaches, and a growing raft of senolytic agents is now available and being assessed in a variety of organ-specific disease models and in clinical trials. Activin A has been reported to impair adipogenesis through the activation of JAK/STAT^35^ and Smad2 signalling pathways^36^ in an autocrine/paracrine manner. Inhibition of Activin A has been shown to boost adipogenesis^36^, reducing lipotoxicity and improving insulin sensitivity^34^. As Activin A was upregulated in *WRN*^−/−^ and *BLM*^−/−^ AP cells, inhibitors targeting this pathway warrant assessment in BS and WS and their *in vivo* models where available. It will also be of great interest to extend findings from this study to other forms of monogenic severe insulin resistance caused by defects in genes involved in DNA damage repair and/or cell control (e.g. *NSMCE2*^50^, *POLD1*^51^, *PCNT*^52^, *POC1A*^53^) to establish if these are unified by a high propensity for senescence in the adipose lineage. This has the potential to define a rare disease target population where trials of senolytics may be of particular value.

## Materials and Methods

### Maintenance of H9 human embryonic stem cell (hESC) line

The human embryonic stem cell line H9 (WiCell) was maintained on Matrigel (Corning)-coated plates at 37°C and 5% CO_2_, in mTeSR1 medium (Stem Cell Technologies). Fresh mTeSR1 medium was applied to the H9 cells every day. Cells were split at a 1:10 ratio approximately every 5 to 7 days when enlarging colonies began to merge.

### CRISPR/Cas9 gene editing

The CRISPR plasmid construct pSpCas9(BB)-2A-Puro (pX459, Plasmid #48139) was obtained from Addgene. Single guide RNA (sgRNA) sequences were designed using a CRISPR design tool (http://crispr.mit.edu) and cloned into the pX459 plasmid. Sequences of sgRNAs are listed in Table S1. H9 cells were dissociated into single cells with Accutase (Stem Cell Technologies). Two million cells were electroporated with 10 µg pX458-sgRNA using the program CA137 on the Lonza Amaxa 4D Nucleofector (Lonza). One day post electroporation, 1 µg/ml puromycin selection was applied to the cells for 48 hours. Cells were then fed fresh mTESR1 media every day until colonies large enough for manual picking and genotyping via Sanger sequencing. Sequences of genotyping primers are listed in Table S4. After targeting, ability of *WRN*^+/+^, *WRN*^−/−^, *BLM*^+/+^ and *BLM*^−/−^ ESCs to differentiated into all 3 germ layers was verified as described by Vallier *et al*. 2009^54^.

### Sister chromatid exchange (SCE) assay

*BLM*^+/+^ and *BLM*^−/−^ ESCs were grown to 80% confluence before they were treated with 10 μM BrdU (Thermofisher) for 48 hours, after which mitotic arrest was induced with 150 ng/ml Colcemid (Gibco) for 30 minutes. Cells were then incubated in 75 mM KCl for 15 minutes at 37°C before fixation in a solution of 3 parts methanol (Sigma-Aldrich) to 1 part glacial acetic acid (Sigma-Aldrich). The cell suspension was dropped onto pre-chilled slides, counterstained with 0.1 mg/ml acridine orange (Molecular Probes), and mounted in Sorenson Buffer, pH 6.8 (0.1 M Na_2_HPO_4_ (Sigma-Aldrich), 0.1 M NaH_2_PO_4_ (Sigma-Aldrich)). Chromatids were visualized using the Zeiss LSM 510 Meta Laser Scanning Microscope (Carl Zeiss) under the FITC filter. SCE events in a metaphase spread was counted and normalized to the total number of chromosomes.

### Differentiation of hESCs into AP cells

Embryoid bodies (EBs) were derived from H9 cells as per Ahfeldt *et al*., 2012^33^. Briefly, H9 colonies were detached from wells and broken into clumps of 5–10 cells in EB formation medium (15% Knockout serum replacement (Thermo Fisher Scientific), 1% GlutaMAX (Thermo Fisher Scientific) in DMEM (Sigma-Aldrich)) supplemented with 4 μM Y-27632 (Sigma-Aldrich). Cell clumps were seeded onto Ultralow attachment plates (Corning) and fed every other day for 5 days after which the EBs were collected and plated onto 0.1% gelatin-coated plates in EB plating medium (10% KnockOut serum replacement (Thermo Fisher Scientific), 1% Glutamax in DMEM (Passage number, P0). Upon reaching 90% confluency, the cellular outgrowths from the EBs were trypsinized and replated onto 0.1% gelatin-coated plates and fed AP medium (15% Knockout serum replacement, 1% GlutaMAX, 2.5 ng/ml bFGF (R&D Systems) in DMEM) every other day (P1). All experiments were performed on P1 AP cells.

### Adipocyte differentiation of AP cells

AP cells were differentiated into adipocytes as described by Ahfeldt *et al*., 2012^33^. Briefly, AP cells were transduced with lenti-PPARγ2 viruses (described in Chen *et al*., 2017^55^) in the presence of 8 µg/ml polybrene. Adipocyte differentiation medium (15% knockout serum replacement, 0.5% non-essential amino acids (Invitrogen), 1% Glutamax, 1 μM dexamethasone (Sigma-Aldrich), 10 μg/ml insulin (Actrapid, Novo Nordisk), 0.5 μM rosiglitazone (Sigma-Aldrich) in DMEM) supplemented with 1 μg/ml doxycycline (Sigma-Aldrich) was applied to confluent transduced AP cells to induce adipocyte differentiation. Adipocyte differentiation medium containing doxycycline was applied to the AP cells every other day for 21 days after which the cells were maintained in doxycycline-free differentiation medium for a further 7 days.

### Cell proliferation assay

Cells were dissociated to single cells and counted. Two thousand cells were seeded into each well of a 96-well plate in triplicate. Cells were harvested at 0, 2, 4, 6, 8, and 10 days and analyzed using the CyQuant Cell Proliferation assay (Invitrogen) per manufacturer’s guidelines. Fluorescence was measured at 520 nm using the Tecan Infinite M1000 Pro Microplate Reader (Tecan).

### Telomere length measurement

Genomic DNA was extracted using the Gentra PureGene cell kit (Qiagen) according to manufacturer’s instructions. The protocol used for measuring telomere length was developed by Cawthon, 2002^32^. Briefly, qPCR was performed using primers specific for telomeres (T) and single copy gene 36B4 (S). Primer sequences can be found in Table S1. T values were then normalized against S to obtain an index of relative telomere length.

### SA-β-gal staining

AP cells fixed with 4% paraformaldehyde (Thermo Fisher Scientific) were incubated with X-gal solution (40 mM citric acid/Na phosphate buffer (Sigma-Aldrich)), 5 mM K4[Fe(CN)6]0.3H2O (Sigma-Aldrich), 5 mM K3[Fe(CN)6] (Sigma-Aldrich), 150 mM sodium chloride (CIMR), 2 mM magnesium chloride (Sigma-Aldrich) and 1 mg/ml X-gal (Sigma-Aldrich) in distilled water) for 16 hours at 37°C. Cells were washed twice with 1X DPBS. Images were captured at 40X magnification using the Olympus DP20 microscope camera attached to an Olympus CKX41 light microscope.

### mRNA expression analysis

Total RNA was purified from cells using the RNeasy Mini Kit (Qiagen). DNase digestion removed contaminating DNA before first-strand cDNA synthesis with the ImProm-II Reverse Transcription System (Promega). qPCR was undertaken using the ABI PRISM 7900 Sequence Detection System (Applied Biosystems) with each well of a 384-well plate containing a 7 µl reaction volume made up of SYBR Green PCR Master Mix (Applied Biosystems), the appropriate primers (Table S1 and S2, Sigma-Aldrich) and 2 µl cDNA. The sample cycle threshold (Ct) values were then normalized against a standard curve generated with serial dilutions of a neat cDNA standard made up of pooled cDNA samples. An internal housekeeping gene (human HPRT) was used to normalize the expression of the genes of interests.

### Protein expression analysis

Cells were washed in ice-cold DPBS and then lysed in 50–100 μl RIPA buffer (Sigma-Aldrich) supplemented with complete protease inhibitor cocktail (Roche) to extract total protein. Protein was resolved on NuPage 4–12% gradient Bis-Tris minigels (Invitrogen) with 1X MOPS buffer (Invitrogen) and then transferred onto a nitrocellulose membrane at 20 V for 7 to 10 minutes using the iBlot dry blotting system (Invitrogen). 5% powdered skimmed milk or 5% BSA, both diluted in Tris-buffered saline (TBST: 0.05 M Tris, 0.138 M NaCl, 0.0027 M KCl, pH 8.0) with 0.1% Tween-20 (Sigma-Aldrich) were used as blocking buffer. Membranes were incubated overnight at 4 °C with the primary antibodies. Membranes were washed 3 times in TBST and then incubated with the appropriate HRP-linked secondary antibodies. The list of antibodies used in this study can be found in (Table S3). The membrane was then washed 3 times in TBST before the protein was interest was visualized using the EMD Millipore Immobilon Western Chemiluminescent HRP Substrate (ECL) (Millipore) and the ChemiDoc (Biorad).

### Immunostaining

Cells were fixed in 4% neutral-buffered formaldehyde (Sigma-Aldrich) and permeabilized and blocked in 10% donkey serum (Sigma-Aldrich) in 0.1% Triton X-100 in DPBS (PBST) for 20 minutes at RT. Cells were incubated with primary antibodies diluted in 1% donkey serum in PBST for 1 hour at RT before they were washed 3 times in DPBS. Appropriate Alexa-fluor-conjugated secondary antibodies diluted 1:1000 in 1% donkey serum in DPBS were applied to the cells for 30 minutes in the dark at RT. Antibodies used in this study are listed in Table S3. Cells were then washed 3 more times in DPBS. DAPI was diluted 1:10,000 in DPBS, added to the cells and incubated for 2 minutes at RT in the dark. Cells were washed a further 3 times with DPBS and then imaged using the EVOS FL imaging system (Life Technologies).

### Flow cytometry

AP cells were incubated in 0.125% trypsin-EDTA (Sigma-Aldrich) for 1 minute at 37 °C to detach them before resuspension in AP medium and transfer into a 15 ml falcon tube. 1 × 10^6^ cells were transferred into each round-bottom polystyrene tube (BD Biosciences), washed once in fluorescence-activated cell sorting (FACS) buffer made up of 0.005 g/ml bovine serum albumin (BSA) (Sigma-Aldrich) in DPBS and then incubated with PE-labeled antibodies against CD73 (BD Biosciences) for 30 minutes in the dark at RT. Cells were pelleted by centrifuging at 1300 rpm for 3 minutes at RT and washed with FACS buffer. The cell pellet was resuspended in 100 μl 1X DPBS and analysed on a BD FACSCalibur flow cytometer with the percentage of CD73-expressing cells determined by acquiring 10,000 events per cell type. Data were analyzed using Flowing software developed by the Turku Center for Biotechnology (Finland).

### Oil Red O staining

Differentiated AP cells were fixed with 10% neutral-buffered formalin solution (Sigma-Aldrich) for 10 minutes at RT at day 28 then rinsed twice in 60% isopropanol. Freshly prepared Oil Red O working solution (6 parts 0.25% Oil Red O in isopropanol: 1 part 60% isopropanol: 3 parts water) was applied to cells for 30 minutes at RT. Excess Oil Red O stain was removed with 60% isopropanol washing before image acquisition.

### Statistical analysis

The two-tailed Student’s T test was used to test for statistical significance where only a single parameter was compared between two groups. A p value of <0.05 was considered statistically significant. All error bars indicate mean + /− standard deviation (SD). All statistical analyses were performed using GraphPad Prism 6.0 (GraphPad Software Inc., San Diego, CA).

## Supplemetary information

Supplemetary information.
