# Supplementary material for: Human pluripotent stem cell-based models suggest preadipocyte senescence as a possible cause of metabolic complications of Werner and Bloom Syndromes
Source: Sci Rep. 2020 May 4;10:7490. doi: 10.1038/s41598-020-64136-8 (PMC7198505; doi:10.1038/s41598-020-64136-8)
Supplement: Supplementary file 1 — Supplemetary information. [file 41598_2020_64136_MOESM1_ESM.pdf]

Human pluripotent stem cell-based models suggest preadipocyte senescence as a possible cause of metabolic complications of Werner and Bloom Syndromes

Kim Jee Goh[1], Jian-Hua Chen[1,2], Nuno Rocha[1,2] and Robert K. Semple[3,1,2*]

[1] The University of Cambridge Metabolic Research Laboratories, Wellcome Trust-MRC Institute of Metabolic Science, Cambridge, UK.
[2] The National Institute for Health Research Cambridge Biomedical Research Centre, Cambridge, UK.
[3] Centre for Cardiovascular Science, Queen's Medical Research Institute, University of Edinburgh, Edinburgh, UK.

Supplementary information

Supplementary Figure S1

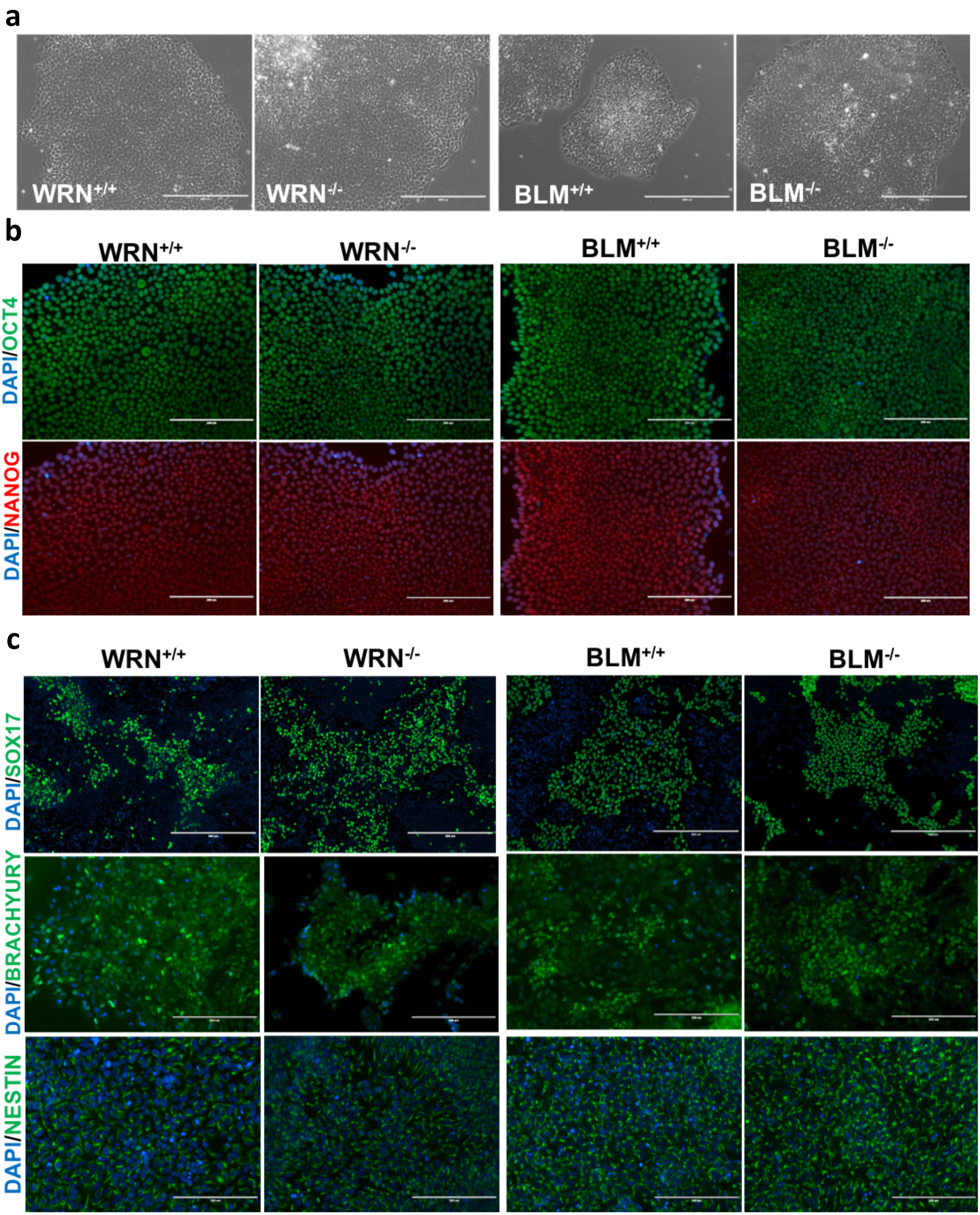

Supplementary Figure S1. WRN$^{+/+}$, WRN$^{-/-}$, BLM$^{+/+}$ and BLM$^{-/-}$ ESCs are viable and pluripotent.

a) Brightfield images of WRN$^{+/+}$, WRN$^{-/-}$, BLM$^{+/+}$ and BLM$^{-/-}$ H9 cells showing normal ESC morphology. Scale bars are 200 µm.

b) WRN$^{+/+}$, WRN$^{-/-}$, BLM$^{+/+}$ and BLM$^{-/-}$ ESCs immunostained for pluripotency markers OCT4 and NANOG. Scale bars are 400 µm.

c) WRN$^{+/+}$, WRN$^{-/-}$, BLM$^{+/+}$ and BLM$^{-/-}$ ESCs differentiated into the 3 germ layers endoderm, mesoderm and neurectoderm and immunostained for the respective markers SOX17, BRACHYURY and NESTIN. Scale bars indicate 400 µm.

Supplementary figure S2

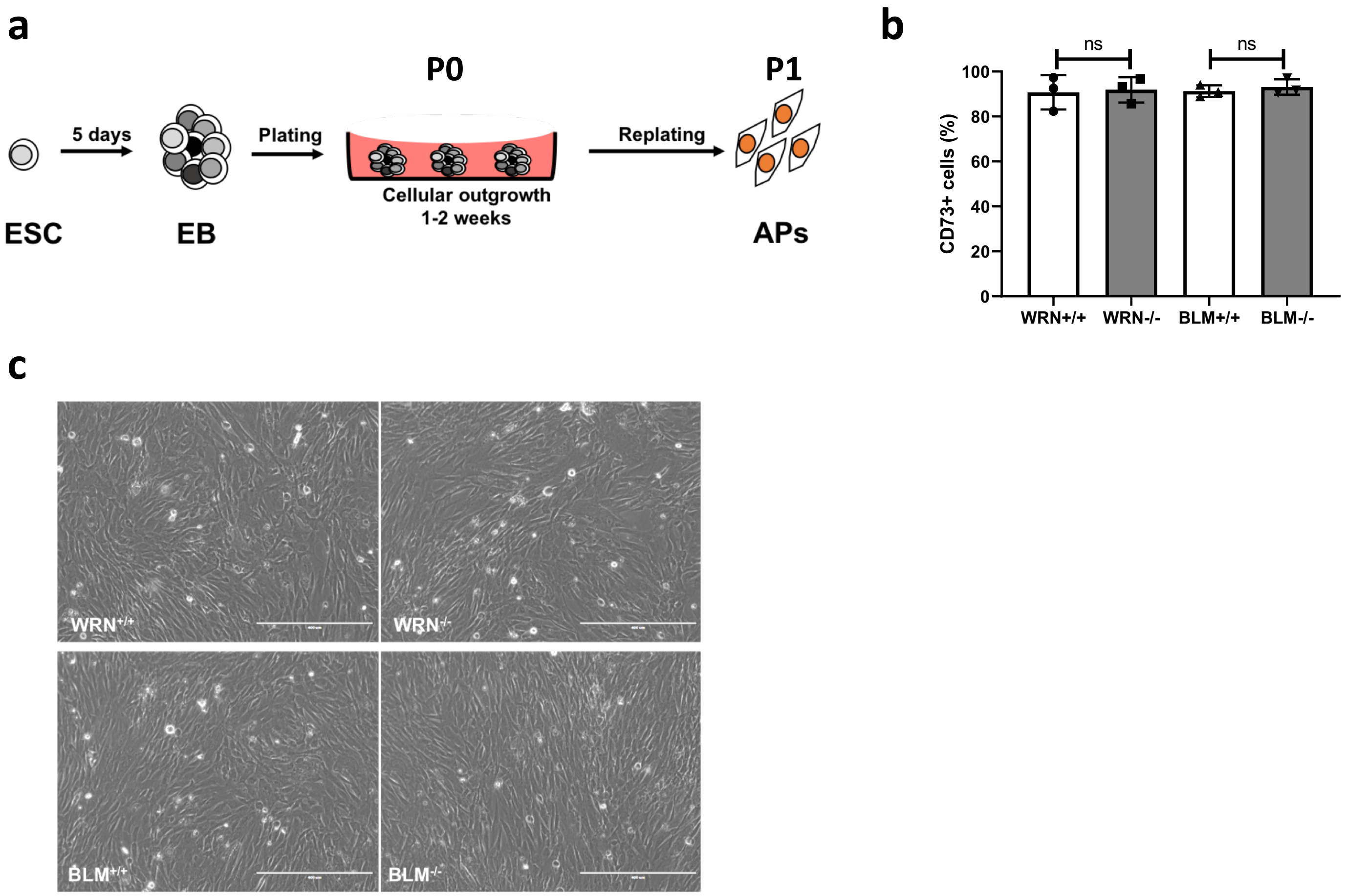

Supplementary Figure S2. Differentiation of $WRN^{+/+}$, $WRN^{-/-}$, $BLM^{+/+}$ and $BLM^{-/-}$ ESCs into adipocyte precursor (AP) cells.

a) Protocol for differentiating human ESCs into adipocyte precursors. P0 and P1 indicate passage numbers.
b) AP differentiation efficiencies as analysed via flow cytometry using AP cell surface marker CD73. n=3. ns, not statistically significant. t test.
c) Brightfield images of AP cells derived from $WRN^{+/+}$, $WRN^{-/-}$, $BLM^{+/+}$ and $BLM^{-/-}$ ESCs. Scale bars are 400 µm.

Supplementary figure S3

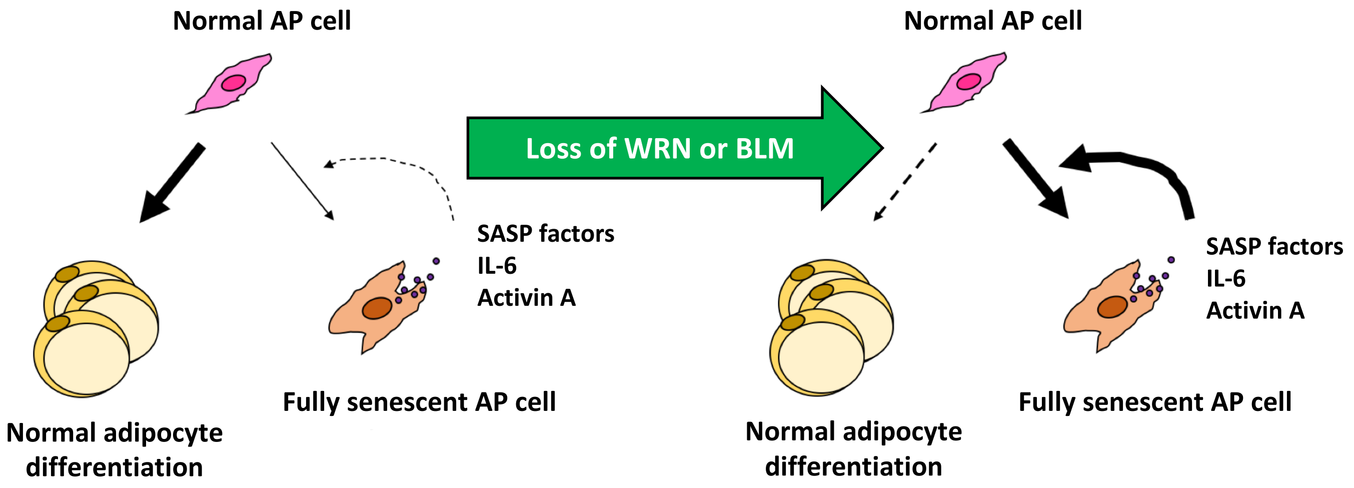

Supplementary figure S3. Overview of how premature senescence in AP cells induced by loss of functional WRN or BLM could establish a positive feedback loop of senescence, ultimately leading to diminished adipose tissue mass and/or function. Loss of WRN or BLM in AP cells sensitises the cells to senescence triggers, initiating the senescence program. Senescent AP cells are unable to efficiently differentiate into mature adipocytes. SASP factors released by these senescent AP cells can act on adjacent non-senescent cells to perpetuate the senescent phenotype, at the same time creating this positive feedback loop of senescence leading to a progressive loss of adipose tissue mass and/or function, which can contribute to metabolic dysfunction.

Supplementary figure S4

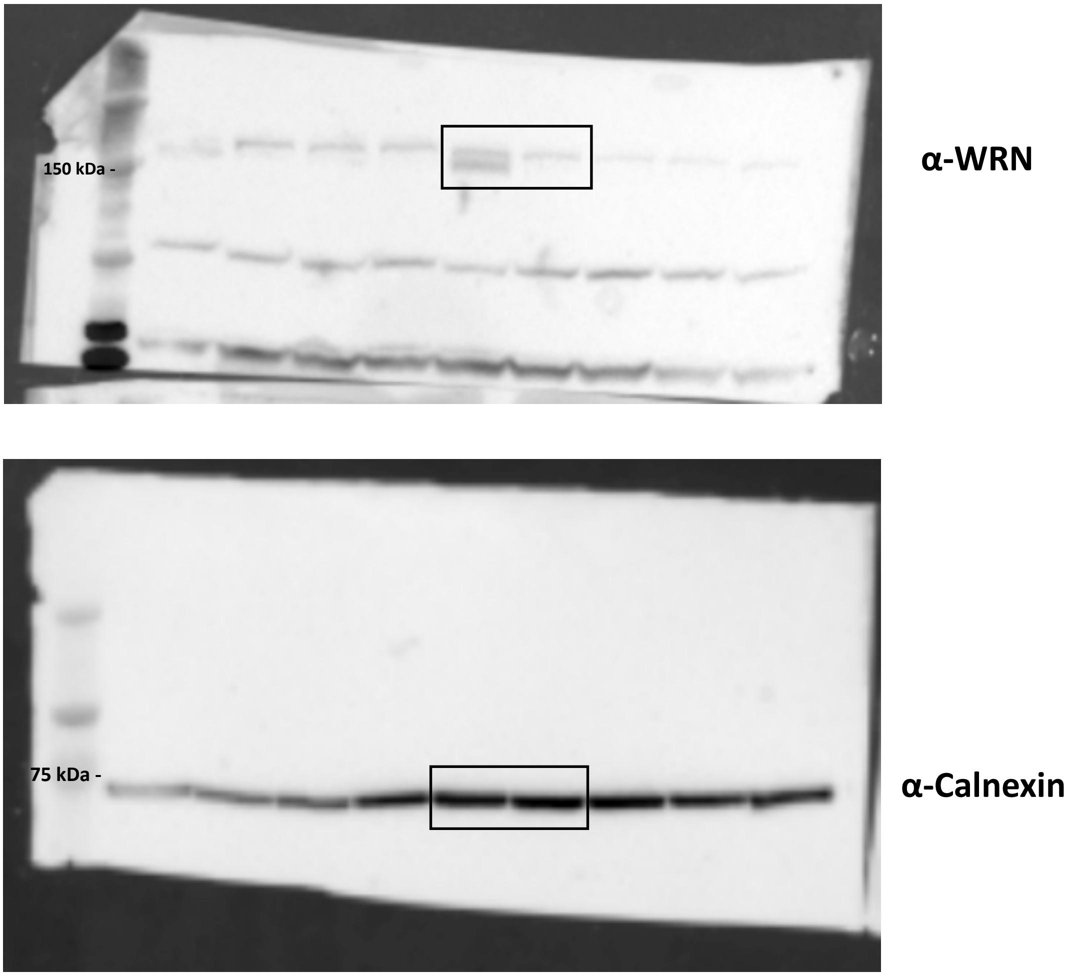

Supplementary figure S4. Full-length Western blots. The boxed regions were presented in Figure 1C.

Table S1 – List of qPCR primer sequences

Name	Sequence (5' to 3')
Activin A F	CCTCGGAGATCATCACGTTTG
Activin A R	GGCGGATGGTGACTTTGGT
Adiponectin F	GATGAAGTCCTGTCTTGGAAGG
Adiponectin R	CAGCACTTAGAGATGGAGTTGG
C/EBPα F	CCACGCCTGTCCTTAGAAAG
C/EBPα R	CCCTCCACCTTCATGTAGAAC
DKC1 F	ACAGGGTGAAGAGTTCTGGCACAT
DKC1 R	TGAAGGTGAGGCTTCCCAACTCAA
FABP4 F	TCATGAAAGGCGTCACTTCC
FABP4 R	GCTTGCTAAATCAGGGAAAACA
HPRT F	TGACACTGGCAAAACAATGCA
HPRT R	GGTCCTTTTCACCAGCAAGCT
IL-6 F	ACTCACCTCTTCAGAACGAATTG
IL-6 R	CCATCTTTGGAAGGTTCAGGTTG
IL-8 F	ACTGAGAGTGATTGAGAGTGGAC
IL-8 R	AACCCTCTGCACCCAGTTTTC
NANOG F	TTTGTGGGCCTGAAGAAAACT
NANOG R	AGGGCTGTCCTGAATAAGCAG
Endogenous PPARG2 F	GCAGGAGATCTACAAGGACTTG
Endogenous PPARG2 R	CCCTCAGAATAGTGCAACTGG
TERC F	CTAACCCTAACTGAGAAGGGCGTA
TERC R	GGCGAACGGGCCAGCAGCTGACATT
TERT F	TGTGCACCAACATCTACAAG
TERT R	GCGTTCTTGGCTTTCAGGAT
Tel F	GGTTTTTGAGGGTGAGGGTGAGGGTGAGGGTGAGGGT
Tel R	TCCCGACTATCCCTATCCCTATCCCTATCCCTATCCCTA
36B4 F	CAGCAAGTGGGAAGGTGTAATCC
36B4 R	CCCATTCTATCATCAACGGGTACAA
WRN sgRNA F	AGCATCGTAACTATACACAATGG
WRN sgRNA R	CCATTGTGTATAGTTACGATGCT
BLM sgRNA F	GATGTGATTTGCATCGATGATGG
BLM sgRNA R	CCATCATCGATGCAAATCACATC

Table S2 – List of Taqman probes

	Company	Catalog No.
BLM	Applied Biosystems	Hs00172060_m1
P16	Applied Biosystems	Hs00923894_m1
P21	Applied Biosystems	Hs00355782_m1
WRN	Applied Biosystems	Hs01087915_m1

Table S3 – List of antibodies

Target protein	Species	Application	Company	Catalog No.	Dilution
BLM	Rabbit	WB	Santa Cruz Biotechnology	sc-7790	1:200
BLM	Goat	WB	Abnova	PAB27648	1:200
WRN	Rabbit	WB	Santa Cruz Biotechnology	sc-5629	1:200
CALNEXIN	Rabbit	WB	Abcam	ab22595	1:5000
GFP	Rabbit	WB	Abcam	ab290	1:5000
NANOG	Goat	IF	R&D Systems	AF1997	1:100
OCT4	Mouse	IF	Santa Cruz Biotechnology	sc-5279	1:100
BRACHYURY	Goat	IF	R&D Systems	AF2085	1:100
NESTIN	Mouse	IF	Abcam	ab22035	1:100
SOX17	Goat	IF	R&D Systems	AF1924	1:200

Table S4 – List of genotyping primer sequences

Gene	Primer ID	Sequence (5’ to 3’)
WRN	exon 3_1 F	CAGCCGGTCTTCAGCATTTT
WRN	exon 3_1 R	AACAGAGCCGATCATAGCCA
WRN	exon 3_2 F	GCCGGTCTTCAGCATTTTAAG
WRN	exon 3_2 R	ACACACAACAGAGCCGATCA
WRN	exon 3_3 F	CCAGCCGGTCTTCAGCAT
WRN	exon 3_3 R	CGCCTGGCCTCTAATGTTTA
BLM	exon 3_1 F	GTGAACCTCTACCCAACACC
BLM	exon 3_1 R	ATGCAAAGCTGTGGACAAGG
BLM	exon 3_2 F	CCCAACACCACAAATCAGCA
BLM	exon 3_2 R	ACCTCAGAGAATGCAAAGCTG
BLM	exon 3_3 F	ACCTCTACCCAACACCACAA
BLM	exon 3_3 R	AGAATGCAAAGCTGTGGACA
